# How Does the Social Support Influence Junior College Students’ Occupational Identity in Pre-school Education?

**DOI:** 10.3389/fpsyg.2022.884606

**Published:** 2022-06-30

**Authors:** Jie Huang, Tianqi Qiao, Zhanmei Song, Jingfeng Yan

**Affiliations:** School of Education, Wenzhou University, Wenzhou, China

**Keywords:** achievement motivation, subjective wellbeing, multiple mediating effect, social support, individual occupational identity

## Abstract

**Objective:**

This study aimed to investigate the multiple mediating effects of achievement motivation and subjective wellbeing between social support and individual occupational identity.

**Methods:**

Questionnaire method was used in this study. 565 junior college students majoring in pre-school education were tested by social support scale, achievement motivation scale, subjective wellbeing scale, and occupational identity scale.

**Results:**

(1) There isn’t significant relationship between perceptions of social support and individual occupational identity. (2) Achievement motivation and subjective wellbeing individually play a mediating role between social support and individual occupational identity. (3) Achievement motivation and subjective wellbeing play a chain mediating effect between elf-efficacy and individual occupational identity.

**Conclusion:**

Social support can indirectly predict professional identity of pre-school “would-be teachers” through the mediating effect of achievement motivation, subjective wellbeing and the chain mediating effect of achievement motivation and subjective wellbeing.

## Introduction

Fully implementing the guiding principle of the fifth plenary session of the 19th Central Committee of Community Party of China (CPC) and the Opinion of the Central Committee of the CPC and the State Council on Deepening the Comprehensive Reform of Teaching Staff for a New Era, the Ministry of Education set The Teachers’ Occupational Capability Standard of Pre-school Education Teachers (Trial) in April, 2021. It suggests that students majoring in pre-school education should cultivate their sentiments of education, and takes occupational identity as an important part.

Teacher occupational identity refers to a teacher’s positive perception and positive evaluation of his or her occupation (Beijaard et al., 2004). As the driving force and key factor of teachers’ professional development, it motivates the external behavior of the subject and determines the attitude of teachers toward the profession to promote teachers’ professional development (Hong, 2010). However, kindergarteners’ occupational identity refers to a mental state in which pre-school teachers have subjective inner acceptance of their own pre-school education career, so as to consciously serve the pre-school education industry (Wang et al., 2014). Although research has showed that the occupational identity of interns in pre-school education is generally above the average level (Gezi and ABD Razak, 2021), in fact, pre-school teachers have high work intensity, heavy tasks and many link requirements, while poor pay and low social status. This series of imbalance factors, to some extent, not only restrict the occupational identity of pre-school education students, but also affect the healthy development of pre-school education.

At present, pre-school teachers in China are mainly trained by infant normal colleges and institute of education or related disciplines in comprehensive universities. Education system of pre-school teachers is opening up and forming a multi-layered pre-school teacher training system. The educational level of pre-school teachers has also been transformed from “junior college + senior high school” to “junior college + undergraduate.” However, according to the education statistics released by the Ministry of Education in 2020, the master degree of full-time pre-school teachers in China accounted for about 0.222%, and that of teachers with bachelor’s degree accounted for about 26.252%. Teachers with junior college degrees accounted for 58.54%, and those with high school degrees or below accounted for 14.986%. Although compared with previous years, the group of pre-school full-time teachers with bachelor’s degree has increased, but the overall educational level is still dominated by junior college, which will not be fundamentally changed in a short time.

Therefore, how to effectively improve the occupational identity level of pre-school education students in the school training will have a very important impact on the development of pre-school education in China. As a reserve army of pre-school teachers, the establishment of occupational identity of pre-school students will directly affect their learning quality and education quality in the future (Gezi and ABD Razak, 2021). Studies have found that occupational identity affects pre-school teachers’ professional happiness and job satisfaction (Butakor et al., 2020). In the absence of occupational identity, it will lead to role imbalance and role conflict, even produces behavioral biases (Peng et al., 2020). Therefore, it is necessary to explore the influencing factors and mechanism of occupational identity of pre-school education college students to carry out effective intervention.

## Theoretical Basis and Hypothesis

### The Influence of Perceived Social on Occupational Identity

A large number of empirical studies have found that social factors such as the support of teachers’ family and friends and the outside world’s views on teachers’ profession have an important influence on the level of teachers’ professional identity (Chen et al., 2020). Stable interpersonal relationships and good social sources can improve their professional identity (Zhao and Zhang, 2017), that is, social support can positively predict individual’s occupational identity. First, the direct effect model of social support (Kawachi and Berkman, 2001) suggests that social support networks can directly lead to positive psychological states, including sense of purpose, belonging and security, and self-identification. Specifically, when individuals have a positive psychological state, they will be motivated to work and produce a sense of identity to the occupation they are engaged in, which is also beneficial to their mental health. According to the theory of job demand-resource model (Bakker et al., 2005), when individuals have independent and timely feedback, social support and high-quality relationship between superiors and subordinates at work, they will make full use of work resources, devote themselves to work better, complete work objectives and recognize their occupation.

*Hypothesis 1*: Social support can positively influence the professional identity of pre-school “to-be teachers.”

### Mediating Role of Achievement Motivation

How does social support affect individual occupational identity? Previous studies on the relationship between these two are mainly analyzed from the perspective of individual factors and social factors, but there are relatively few studies on the mechanism of individual motivation level and psychological feeling in the relationship between these two. In addition, are there any other factors playing a role in the relationship between professional identity and social support of pre-school students? Therefore, this study introduced a chain mediation model constructed by achievement motivation and subjective wellbeing, and analyzed its chain mediation effect, so as to explore the internal mechanism between social support and occupational identity of pre-school “to-be teachers.”

Achievement motivation refers to an internal driving force that individuals are willing to do what they consider important and valuable and strive to achieve perfection (Ball, 1982). According to Atkinson, there are two different motivational tendencies in every person: the pursuit of success and the avoidance of failure. The motivation to pursue success is the approach to the achievement goal, while the motivation to avoid failure is the avoidance of the achievement goal due to some negative factors (Carver, 2006). The approach motivation will make individuals experience more positive emotions (Carver et al., 2000) and will be more likely to have good relationships (Mehrabian, 1994); Avoidant motivation does the opposite. As a necessary condition for individual growth, social support is also an important place for human socialization. In today’s society, social support comes from a wide range of sources, such as family, lovers, children, parents, friends and various social organizations (Alsubaie et al., 2019). With such a wide range of sources, the strength of the sense of social support has a certain impact on the psychological situation of college students (Alsubaie et al., 2019). Domestic scholars also confirmed that social support level has a significant positive predictive effect on achievement motivation in the study of college students’ achievement motivation (Ahmed et al., 2010). According to the achievement motivation theory (Anderman, 2020), when the motivation to pursue success is stronger than the motivation to avoid failure, individuals will take positive actions. Moreover, if individuals live in an environment where there are no parents or friends to encourage their achievements or progress, that is, the environment does not provide minimal support for their efforts. Individual achievement motivation level will not be improved. According to the theory of achievement need (Schüler et al., 2010), high achievement motivation requires individuals to desire to do things more perfectly, improve work efficiency, and achieve greater success. Moreover, individuals mainly focus on work itself, and are more likely to develop professional identity with work. Exploration of achievement motivation is the “way” of exploration education, good achievement motivation can stimulate and maintain students’ good professional identity (McClelland, 1965). Research also proved that the level of achievement motivation can significantly predict college students’ professional identity (Shu and Hong, 2012).

*Hypothesis 2*: Achievement motivation is the mediating variable of social support influencing individual professional identity.

### Mediating Role of Subjective Wellbeing

In addition, subjective wellbeing mainly focuses on individual subjective feelings, mostly focusing on the evaluation of life satisfaction and happiness (Diener, 1984). In the field of psychology, it is generally believed that social support has an extremely important influence on subjective wellbeing. When individuals have good social support, their life satisfaction and positive emotions are relatively high, while their negative emotions are relatively low (Ji et al., 2019). The stress buffering model of social support suggests (Kawachi and Berkman, 2001) that social support can prevent or modulate responses to stressful events that are harmful to health. The perception of social support in the face of a stressful event may lead to a positive evaluation of the situation, thereby preventing subsequent negative emotional and behavioral reactions. That is, when the level of social support is high, individuals are more likely to have positive emotions. At the same time, domestic and foreign studies have confirmed that social relations have an important impact on subjective wellbeing. Zalewska (2018) believes that in collectivist cultures (such as China and Japan, etc.), individuals must always maintain coordination with others (such as obeying others’ needs and expectations), which is conducive to their experience of higher subjective wellbeing. In other words, for individuals, good social relationships can improve their happiness. Domestic scholars have also confirmed that, for college students, major social relationships (family relationship, friend relationship, romantic relationship, and roommate relationship) are indeed important factors influencing their subjective wellbeing (Heintzelman and Diener, 2019). Studies have found that professional identity is significantly positively correlated with subjective wellbeing (Jue and Ha, 2018). According to social identity theory (Hogg, 2018), in order to establish and maintain a positive image of oneself, individuals need to positively identify themselves. As an important part of self-identity, individuals’ positive evaluation of themselves is conducive to the formation of professional identity and the sense of the significance of work. In addition, studies reveal that individuals who identify with their careers tend to associate themselves with their careers, so that they can obtain a sense of meaning and psychological continuity of work (Gertsog et al., 2017), thus improving job satisfaction.

*Hypothesis 3*: Subjective wellbeing is another mediating variable of social support affecting professional identity.

### Multiple Mediating Role of Achievement Motivation and Subjective Wellbeing

In addition, the study also found that the level of achievement motivation affects people’s happiness, and the pursuit of success and the avoidance of failure have different effects on happiness. According to the goal theory of subjective happiness (Klug and Maier, 2015), when the needs of individuals are met or the goals are realized, individuals will get positive emotions and then realize their subjective happiness. That is, when an individual is satisfied with the goal at a certain level, his sense of self-efficacy will be improved, and his subjective happiness in this aspect will be improved, and then he will pursue a higher level of subjective happiness. For example, pursuing and maintaining meaningful goals plays an important role in the maintenance and development of individual happiness (Klug and Maier, 2015). Approach motivation is related to higher subjective wellbeing. However, individuals with strong motivation to avoid are associated with lower subjective wellbeing (Tian et al., 2017). A large number of domestic and foreign studies have found that achievement motivation can predict subjective wellbeing.

*Hypothesis 4*: Achievement motivation and subjective wellbeing play a continuous intermediary role in the associations between social support and occupational identity.

**FIGURE 1:**
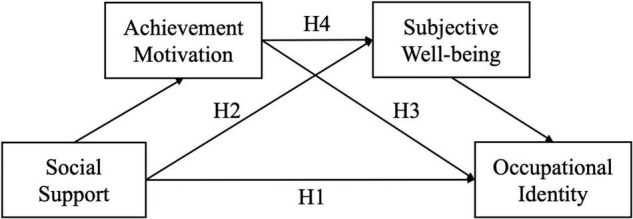
Hypothesized model.

Hypothesized model are shown in Figure 1. To sum up, this study intends to explore “how social support affects” the professional identity of pre-school prospective teachers, focusing on analyzing the chain mediating role of achievement motivation and subjective wellbeing in the relationship between social support and professional identity of pre-school prospective teachers.

## Materials and Methods

### Participants

The participants were recruited from a University located in Wenzhou, Zhejiang Province, China. Questionnaires were distributed to 627 Chinese students of pre-school education specialized classes, and 565 valid questionnaires were collected, giving a response rate of 90.11%.

Among them, 545 (96.5 percent) were female and 20 (3.5 percent) were male. 460 freshmen (81.4%) and 105 sophomores (18.6%); There were 127 only children (22.5%) and 438 non-only children (77.5%).

### Measures

To ensure the accuracy of empirical research, this study references important literature published locally and abroad, and selects maturity scales with high reliability and validity.

### Social Support

Measurement of social support used the Social Support Appraisals (SS-A) Scale, which was originally compiled by Vaux et al. (1986). The scale has been proved to have good reliability and validity in China (Chi et al., 2007). There were 20 items (e.g., “My family cares a lot about me”), in which 7 questions measured family support, 7 questions measured friend support, and 6 questions measured general others support. All items are scored with 5 points. The average score of all items represents the individual’s social support. The higher the score is, the better the social support is. The Cronbach’s alpha coefficient of the scale in this study was 0.848, indicating that the scale has good reliability.

### Occupational Identity

Based on the Professional Identity Questionnaire for Pre-school Teachers compiled by Wang (2009), 14 items includes professional cognition, professional needs, professional emotion and professional will. Each item is scored with Likert 5 points, and the score ranges from 1 point for “strongly disagree” to 5 points for “strongly agree.” The higher the score of occupational cognition, occupational need, occupational emotion and occupational will, the greater the identification in this aspect. The Cronbach’s α coefficient of the total questionnaire and four sub-questionnaires ranged from 0.50 to 0.79. The results show that the overall Cronbach’s α coefficient of the scale is 0.86, and the scale has good reliability.

### Achievement Motivation

The achievement motivation questionnaire was measured by the Achievement Motivation Scale (AMS) (Ye and Hagtvet, 1992). There are 30 items in total, which can be divided into two dimensions, namely, the motivation to pursue success and the motivation to avoid failure. Four points were used (1 very inconsistent to 4 very consistent). Achievement motivation score is the score of pursuing success minus the score of avoiding failure, the higher the score, the stronger the achievement motivation. The results show that the Cronbach’s α coefficient of the scale is 0.86, and the scale has good reliability.

### Subjective Wellbeing

To measure subjective wellbeing, we uses Subjective Wellbeing of Chinese Urban Residents (SWBS-cc20) revised by Xing (2003). The scale contains 20 items from 10 dimensions, including contentment and abundance experience, mental health experience, social confidence experience, growth and progress experience, target value experience, self-acceptance experience, physical health experience, mental balance experience, interpersonal adaptation experience and family atmosphere experience. Each item is scored with Likert 6 points. Points range from one for strongly disagree to six for strongly agree. The results show that the Cronbach’s α coefficient of the scale is 0.89, and the scale has good reliability.

### Control Variables

The study controls demographic variables, such as gender and age.

## Results

### Common Method Deviation Test

This paper uses Harman’s single factor analysis to evaluate the common source variance. Exploratory factor analysis was performed without rotation. The results showed that there are 17 factors with eigenvalues greater than 1, and the variation explained by the first factor is 19.35%, which is less than the critical standard of 40%. Therefore, no common method bias effect was observed between the measured variables.

### Descriptive Statistics and Correlations

The mean, standard deviation, and correlation coefficient of latent variables were statistically analyzed using SPSS 22.0. As shown in Table 1, the mean and standard deviation of each variable were within the acceptable range. According to the correlation coefficient between variables, a significant correlation exists between social support, occupational identity, achievement motivation, and subjective wellbeing. The results of descriptive statistics and related analysis preliminarily illustrate the relationship between variables, providing a basis for further data analysis.

**TABLE 1:** Descriptive statistics and correlations analysis (*n* = 565).

	Variable	*M*	SD	1	2	3	4
1	Social support	59.610	6.850	–			
2	Occupational identity	42.330	7.878	0.244**	–		
3	Achievement motivation	-3.290	11.791	0.221**	0.326**	–	
4	Subjective wellbeing	79.900	12.982	0.591**	0.417**	0.455**	–

**p < 0.01.

### Social Support and Occupational Identity: Mediating Effect Test

Under the control of gender, grade, and whether he/she is an only child, the SPSS plug-in woven by Hayes (2013) was used to evaluate the 95% confidence interval of the mediating effect of achievement motivation and subjective wellbeing in the impact of social support on occupational identity (Bootstrap sample is 5000).

The results showed that the total effect of social support on occupational identity was significant (β = 0.246, *p* < 0.001), supporting H1. After including the mediating variables, social support not only significantly positively predicted the achievement motivation of pre-school “to-be teachers” (β = 0.221, *p* < 0.001), but also significantly and positively predicted the subjective wellbeing of pre-school “to-be teachers” (β = 0.513, *p* < 0.001). However, the direct effect of social support on occupational identity was not significant (β = 0.008, *p* = 0.860). Achievement motivation can not only significantly and positively predict the subjective wellbeing of pre-school “to-be teachers” (β = 0.345, *p* < 0.001), but also can significantly positively predict the occupational identity of pre-school “to-be teachers” (β = 0.161, *p* < 0.001). Besides, Subjective wellbeing can significantly and positively predict the occupational identity of pre-school “to-be teachers” (β = 0.343, *p* < 0.001) (Shown in Table 2).

**TABLE 2:** Regression analysis of variable relationships in the model.

Regression equation		Fit index			Regression coefficient significance	
		
Outcome variable	Predictor variable	R	R^2^	*F*	*B*	*t*
Occupational identity		0.269	0.072	10.904***		
	Gender				0.352	1.568
	Grade				0.202	1.921
	One child				–0.118	–1.194
	Social support				0.246	6.038***
Achievement motivation		0.238	0.057	8.388***		
	Gender				0.428	1.890
	Grade				0.080	0.754
	One child				–0.039	–0.393
	Social support				0.221	5.388***
Subjective wellbeing		0.680	0.462	95.939***		
	Gender				–0.120	–0.696
	Grade				–0.090	–1.116
	One-child				0.058	0.769
	Achievement motivation				0.345	10.792***
	Social support				0.513	16.110***
Occupational identity		0.458	0.209	24.617***		
	Gender				0.273	1.312
	Grade				0.210	2.160
	One child				–0.127	–1.388
	Achievement motivation				0.161	3.782***
	Subjective wellbeing				0.343	6.688***
	Social support				0.008	0.172

***p < 0.001. All variables of the model are standard scores.

**FIGURE 2:**
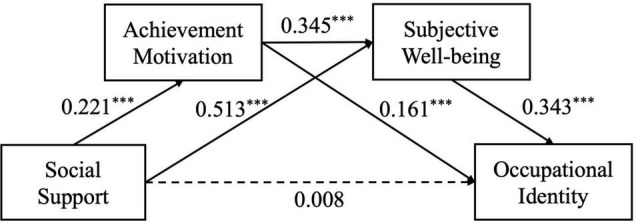
Intermediary model of social support and occupational identity. ***p < 0.001.

The chain multiple mediation effect was tested. The results has showed that the 95% confidence interval of the mediating effect was multi-mediation effect of achievement motivation and subjective wellbeing was tested significantly. The results are shown in Figure 2 and Table 3.

**TABLE 3:** Summary of the indirect effects.

Intermediate path	Effect value	Boot standard error	Relative mediating effect	95% confidence interval	
				Lower limit	Upper limit
Total indirect effect	0.238	0.034		0.176	0.305
SS→AM→OI	0.036	0.014	15.006%	0.009	0.066
SS→SW→OI	0.176	0.030	74.023%	0.117	0.239
SS→AM→SW→OI	0.026	0.007	11.013%	0.013	0.044

*SS, social support; AM, achievement motivation; SW, subjective wellbeing; OI, occupational identity.*

Social support → Achievement motivation → Occupational identity mediating effect is 0.036, 95% confidence interval is (0.009, 0.066), excluding 0, supporting H2, and the mediating effect accounted for 15.006%. Social support → Subjective wellbeing → Occupational identity, the mediating effect is 0.176, the 95% confidence interval is (0.117, 0.239), excluding 0, supporting H3, and the mediating effect accounted for 74.023%. Social support → Achievement motivation→ Subjective wellbeing → Occupational identity chain multi-mediating effect is 0.026, 95% confidence interval (0.013, 0.044), supporting H4, and the mediating effect accounted for 11.013%.

## Discussion

The results of this study show that achievement motivation and subjective wellbeing each independently mediate the relationship between social support and occupational identity of pre-school “to-be teachers.” First, achievement motivation is an important mediator for social support to influence the occupational identity of pre-school “to-be teachers.” On the one hand, the level of individual social support is significantly positively correlated with achievement motivation, which is consistent with previous research results (Morales Navarro et al., 2020), and in line with the viewpoint of achievement motivation theory (Anderman, 2020). That is, the higher the level of social support, the more likely individuals are to take positive actions and the stronger the motivation to pursue success will be. On the other hand, achievement motivation can positively predict the occupational identity of pre-school “to-be teachers,” which is consistent with previous research results (Hong et al., 2018), and in line with the achievement need theory, that is, high achievement motivation will make individuals improve their work efficiency, focus their attention on the work itself, form positive cognition of work, and effectively improve the level of individual professional identity. Second, the study found that social support can also affect individual occupational identity through the effect of subjective wellbeing. On the one hand, social support can positively predict individual subjective wellbeing, which is consistent with the results of previous studies (Zalewska, 2018) and the view of the stress buffer model of social support (Kawachi and Berkman, 2001). That is, when individuals with high levels of social support respond to various life events, it is more likely to produce positive emotions, so that individuals can form positive and optimistic qualities to face challenges at work. On the other hand, subjective wellbeing has a significant positive predictive effect on occupational identity, which is consistent with previous research results (Jue and Ha, 2018). According to the social identity theory, SWB, as an individual subjective feeling, can help individuals positively evaluate themselves and feel the significance of work, thereby improving individual occupational identity.

In addition, this study also found that the chain mediator composed of “achievement motivation → subjective wellbeing” is also an important way for social support to affect the professional identity of pre-school “to-be teachers.” On the one hand, pre-school “to-be teachers” with high achievement motivation will focus more on the work content of the pre-school education career, hoping to make the work better through their own efforts, rather than affecting the work of others, and have higher sense of responsibility and sense of honor at work that enables individuals not only to be proactive in their daily work, but also to exert their personal abilities when they are in a challenging work environment, stick to their jobs, and handle work with an optimistic and positive attitude. Contradictions that appear in children, and treat each child with a more full and enthusiastic state, which has a significant effect on promoting the level of individual subjective wellbeing, and this result is also consistent with previous research results (Schütz et al., 2019). On the other hand, this finding is consistent with the goal-theoretic view of subjective wellbeing (Klug and Maier, 2015), which argues that individuals receive positive positivity when their needs are met or goals are achieved. Emotion, and then realize the subjective wellbeing of the individual. According to the above point of view, achievement motivation, as a positive internal driving force for individuals, can help individuals to continuously improve their work efficiency in focused work. Besides, individuals with high achievement motivation can help them maintain an optimistic and positive attitude in a challenging work environment, enhance their psychological capital, and treat the future development of pre-school education with enthusiasm and enthusiasm, and improve their subjective wellbeing. Level, and subjective wellbeing is also an important protective factor for individual occupational identity (Watanabe et al., 2019). Therefore, the chain intermediary composed of “achievement motivation → subjective wellbeing” is also an important bridge for social support to affect the professional identity of pre-school “to-be teachers.”

By constructing a chain mediation model, this study answers the question of “how social support affects” the professional identity of pre-school “to-be teachers,” that is, social support can be achieved through the independent mediating effect of achievement motivation and subjective wellbeing and the chain mediating effect of the two. Pre-school “to-be teacher” professional identity. The independent mediating roles of achievement motivation and subjective wellbeing support achievement needs theory and job requirement-resource model theory, which helps us to reveal the impact of social support on the professional identity of pre-school “prospective teachers” from the perspective of individual motivation and emotional state mechanism. In addition, according to achievement needs theory and job requirement-resource model theory, the chain mediating role of achievement motivation and subjective wellbeing is useful for in-depth analysis of these two between social support and pre-school “prospective teacher” professional identity. Also, it indicates that achievement motivation not only mediates the influence of social support on individual occupational identity, but also enhances individual occupational identity by promoting the level of individual subjective wellbeing.

### Limitations and Future Study Directions

In terms of research samples, due to the limitations of the research objects, this study only selected the pre-school education junior college students in comprehensive universities for analysis, and did not collect more targeted data on pre-school education junior college students under the educational background of vocational colleges. Second, considering the dynamic nature of research variables such as occupational identity and achievement motivation that change over time, future research can be considered from the perspective of longitudinal tracking. In addition, the impact of social support on individuals is multifaceted and multidimensional. Future research can increase the dimension of social support research variables and further enrich and develop research models and conclusions. This study only considered mediating factors between social support and individual occupational identity. Therefore, future studies should consider whether there may be other factors that mediate this mechanism and incorporate these factors into the research framework.

## Data Availability Statement

The original contributions presented in the study are included in the article/Supplementary Material, further inquiries can be directed to the corresponding author.

## Author Contributions

JH led the research design, data analysis, and drafted this manuscript. TQ guided the research design and revised the manuscript substantially. ZS revised the section of the analysis and discussion and corrected the entire manuscript. JY analyzed and verified the data in this manuscript. All authors approved the final version.

## Conflict of Interest

The authors declare that the research was conducted in the absence of any commercial or financial relationships that could be construed as a potential conflict of interest.

## Publisher’s Note

All claims expressed in this article are solely those of the authors and do not necessarily represent those of their affiliated organizations, or those of the publisher, the editors and the reviewers. Any product that may be evaluated in this article, or claim that may be made by its manufacturer, is not guaranteed or endorsed by the publisher.
